# Structural basis of conformational transitions in the active site and 80′s loop in the FK506-binding protein FKBP12

**DOI:** 10.1042/BJ20131429

**Published:** 2014-02-28

**Authors:** Sourajit M. Mustafi, Matthew Brecher, Jing Zhang, Hongmin Li, David M. Lemaster, Griselda Hernández

**Affiliations:** *Wadsworth Center, New York State Department of Health Empire State Plaza, Albany, NY 12201, U.S.A.; †Department of Biomedical Sciences, School of Public Health, University at Albany (SUNY), Empire State Plaza, Albany, NY 12201, U.S.A.

**Keywords:** conformational dynamics, FK506-binding protein 12 (FKBP12), linebroadening analysis, NMR, slow exchange, X-ray structure, FKBP, FK506-binding protein, mTOR, mammalian target of rapamycin, RyR, ryanodine receptor

## Abstract

The extensive set of NMR doublings exhibited by the immunophilin FKBP12 (FK506-binding protein 12) arose from a slow transition to the *cis*-peptide configuration at Gly^89^ near the tip of the 80′s loop, the site for numerous protein-recognition interactions for both FKBP12 and other FKBP domain proteins. The 80′s loop also exhibited linebroadening, indicative of microsecond to millisecond conformational dynamics, but only in the *trans*-peptide state. The G89A variant shifted the *trans*–*cis* peptide equilibrium from 88:12 to 33:67, whereas a proline residue substitution induced fully the *cis*-peptide configuration. The 80′s loop conformation in the G89P crystal structure at 1.50 Å resolution differed from wild-type FKBP12 primarily at residues 88, 89 and 90, and it closely resembled that reported for FKBP52. Structure-based chemical-shift predictions indicated that the microsecond to millisecond dynamics in the 80′s loop probably arose from a concerted main chain (ψ_88_ and ϕ_89_) torsion angle transition. The indole side chain of Trp^59^ at the base of the active-site cleft was reoriented ~90^o^ and the adjacent backbone was shifted in the G89P crystal structure. NOE analysis of wild-type FKBP12 demonstrated that this indole populates the perpendicular orientation at 20%. The ^15^N relaxation analysis was consistent with the indole reorientation occurring in the nanosecond timeframe. Recollection of the G89P crystal data at 1.20 Å resolution revealed a weaker wild-type-like orientation for the indole ring. Differences in the residues that underlie the Trp^59^ indole ring and altered interactions linking the 50′s loop to the active site suggested that reorientation of this ring may be disfavoured in the other six members of the FKBP domain family that bear this active-site tryptophan residue.

## Abstract 2

Extensive resonance doubling arises from a *cis*–*trans* peptide transition at Gly^89^, whereas linebroadening appears due to a concerted shift in the neighbouring torsion angles. The active site Trp^59^ ring adopts a perpendicular orientation at a population of 20%.

## INTRODUCTION

The peptide prolyl isomerase FKBP12 (FK506-binding protein 12) was initially characterized for its role in binding the immunosuppressants FK506 and rapamycin. The ternary complex of FKBP12 and FK506 with calcineurin inhibits the enzymatic activity of this protein phosphatase, blocking a key T-cell-activation pathway involved in tissue transplant rejection [1]. The binding of FKBP12 and rapamycin to mTOR (mammalian target of rapamycin) inhibits its role in regulating cell growth and cancer progression [2]. Despite considerable effort directed towards developing active-site inhibitors of mTOR, the only two clinically approved therapeutics for this protein kinase are allosteric inhibitors derived from rapamycin [3].

The challenge of FKBP12 pharmacology is increased markedly by the presence of 22 homologous FKBP domains within the human genome [4]. In addition to the closely homologous FKBP12.6 and the more evolutionarily divergent FKBP13, there are 19 different FKBP domains that are modules within larger proteins. In addition to structural similarities, there is also some degree of functional overlap among these FKBP domain proteins. The mTOR interactions of FKBP12 can be functionally replaced in cellular model systems by the larger FKBP domain proteins FKBP51 and FKBP52 [5]. In a similar fashion, siRNA studies indicate that FKBP12 contributes to ~60% of the FK506-mediated inhibition of calcineurin, whereas the rest of the inhibitory effect is contributed nearly equally by FKBP12.6 and FKBP51 [6].

Although the pharmacological utility of the bacterial macrolides FK506 and rapamycin is well established, the physiological relevance of their interactions is less clear. Despite more than two decades of research, no endogenous small-molecule ligand has been shown to mediate protein–protein interactions for the FKBP domains [7]. Both biochemical [8,9] and genetic [10,11] evidence support the importance of the binding of FKBP12 and FKBP12.6 to the RyR (ryanodine receptor) Ca^2+^ channels in cardiac and skeletal muscle in the regulation of contraction, as well as binding to the RyR channels of pancreatic islet cells for the regulation of insulin secretion [12] and to RyR channels in the central nervous system that are involved in memory processing [13] and in stress-induced cognitive dysfunctions [14]. FKBP12 has also been implicated in the regulation of a number of membrane-bound hormone receptors [15].

The prolyl isomerization activity of FKBP12 has not been shown to participate in any of its protein signalling interactions, and a similar lack of dependence on catalytic activity has been observed for the well-studied glucocorticoid receptor interactions of the closely homologous FKBP domains of FKBP51 and FKBP52 [16]. On the other hand, prolyl isomerization activity does appear to play a role in the association of FKBP12 with aggregated α-synuclein in the Lewy body deposits of patients with Parkinson's disease [17], with the conformationally disordered tau protein in the neurofibrillary tangles of Alzheimer's disease [18] and in the binding to the amyloid precursor protein [19]. FKBP12 has been shown to accelerate the aggregation of α-synuclein *in vitro* [20] and *in vivo* [21].

Recently, we characterized a previously unreported slow conformational transition for FKBP12 in which a minor state population of 12% interchanges with the major conformational state at a rate of ~0.05 s^−1^ at 25°C [22]. Although the primary site of the conformational transition appears to lie at the tip of the 80′s loop, which spans between the last two strands of the central β-sheet, the doubling of the backbone amide resonances that arises from the two slowly interchanging conformations extends not only to residues lining the active-site cleft, but beyond to a number of residues in and surrounding the 50′s loop on the opposite side of the protein. Interestingly, the major state, but not the minor state, of this slow transition also exhibits linebroadening of the amide resonances in the 80′s loop, indicative of conformational exchange in the microsecond to millisecond timeframe. Evidence for conformational flexibility within the 80′s loop gains particular relevance due to the fact that this loop provides a major proportion of the interprotein interactions for each of the four distinct protein–protein complexes that have been structurally determined for FKBP12 [23–26]. The homologous loop has also been shown to provide critical protein-recognition interactions in other FKBP domain proteins [16].

In the present study we have analysed the structural basis of the slow resonance doubling transition of FKBP12 and the more rapid conformational linebroadening transition in the 80′s loop to gain insight into how these effects are propagated through the protein structure. In turn, these studies have led to the characterization of a well-populated conformational transition within the active-site cleft.

## EXPERIMENTAL

### Protein preparation

Genes for the wild-type and G89A and G89P variants of the human FK506-binding protein FKBP12 were synthesized chemically (Genscript) from the wild-type gene sequence, with codon optimization for expression in *Escherichia coli* cells. The genes were cloned into the expression vector pET11a and then transformed into the BL21(DE3) strain (Novagen) for expression. The protein expression and purification procedure for the FKBP12 proteins were carried out as described previously [22,27].

All isotopically labelled samples were prepared via protein expression in minimal medium containing 0.1% ^15^NH_4_Cl as a nitrogen source [28]. For U-^13^C,^15^N-enriched samples, 0.2% [U-^13^C]glucose (Cambridge Isotopes) was substituted for the unlabelled glucose used for preparing the U-^15^N samples. For the selective [^13^C]methyl-labelled samples, 85 mg/l [3-^2^H,4-^13^C]α-oxoisovalerate and 50 mg/l [3-^2^H_2_,4-^13^C]α-oxobutyrate [29] were supplemented into a medium for U-^2^H,^15^N-enriched sample growth as described previously [28].

For the protein samples expressed in ^2^H_2_O medium, 1 mM tris(2-carboxyethyl)phosphine and solid Tris base was added to a solution of the purified protein to obtain a pH value above 9, and the samples were incubated at 25°C for 3 h and then neutralized with solid monobasic sodium phosphate. All protein samples were concentrated via centrifugal ultrafiltration (1000 ***g*** for 30 min at 4°C) and then equilibrated into a pH 6.50 buffer containing 25 mM sodium phosphate, 2 mM DTT and 2 mM tris(2-carboxyethyl)phosphine by a series of centrifugal concentration steps (1000 ***g*** for 30 min at 4°C). For the crystallization trials, the protein sample was neutralized and then equilibrated into 5 mM sodium chloride and concentrated by centrifugal ultrafiltration (1000 ***g*** for 30 min at 4°C).

### X-ray crystallography

Crystals of the C22V,G89P variant of FKBP12 were grown at room temperature (22°C) in hanging drops by microseeding from a crushed crystal of the FKBP C22V,H87V variant [22]. Before microseeding, 2 μl of protein solution at 22 mg/ml concentration was mixed with an equal volume of reservoir solution containing 64% saturated ammonium sulfate, 0.1 M Hepes (pH 7.5) and 4% methyl-2,4-pentanediol, and equilibrated overnight. The crystals belonged to space group *C*2 and had the cell parameters *a*=71.37 Å, *b*=35.86 Å, *c*=41.24 Å and β=96.65°. There was one molecule per asymmetric unit with a crystal solvent content of 48%. Before data collection, crystals were gradually transferred into a reservoir solution containing a higher concentration of ammonium sulfate (up to 80%) at 5% per step, and then flash-cooled under a nitrogen stream at 100 K and stored in liquid nitrogen. Diffraction data were collected at 100 K using an RAxisIV++ detector and an in-house Rigaku microfocus Micromax-007 X-ray generator. These diffraction data were processed and scaled using CrystalClear 1.3.6 (Rigaku). Subsequently, higher-resolution diffraction data were collected at 100 K using beamline X25 of the National Synchrotron Light Source (Brookhaven National Laboratory). These data were processed and scaled using HKL2000 [30]. With the high-resolution structure of FKBP12 [31] (PDB code 2PPN) used as a search model, clear solutions were found with the PHASER molecular replacement program within the PHENIX suite [32]. Structural refinement was carried out using PHENIX and SHELX-97 [33]. Model rebuilding was carried out using Coot [34]. Figures of crystallographic structures were generated using the Chimera software [35].

### NMR spectroscopy

NMR data for the G89A and G89P variants of FKBP12 were collected on a Bruker Avance III 600 MHz and 900 MHz spectrometers at 25°C. Backbone resonance assignments were carried out using standard HNCO [36], HN(CA)CO [36], HNCACB [37] and HN(CO)CACB [38] experiments. The resonance assignments for the wild-type protein have been reported previously [22] (Biological Magnetic Resonance Data Bank entries 19240 and 19241). The *cis*-glycine linkage analysis and the indole ring reorientation analysis were carried out using 3D ^13^C-^13^C-^1^H NOESY and ^13^C-^15^N-^1^H NOESY [39] experiments respectively. FELIX software (Felix NMR) was used for the NMR data processing. Relaxation analysis of the ^15^N indole resonance of Trp^59^ utilized a CSA (chemical shift anisotropy) value of −126 p.p.m. [40].

The initial rate of NOE build-up between an isolated proton pair is proportional to:

(1) $$6J_{ij}\left(2\omega \right)-J_{ij}\left(0\right)$$

where *ω* is the ^1^H Larmor frequency and *J_ij_*(X) are the com-ponents of the spectral density function. If the correlated internal motion of the two nuclei dissipates rapidly as compared with the rate of global molecular tumbling, the internal motion correlation function can be factored out as a generalized order parameter (*S*) [41] which for a three state methyl jump model can be represented as:

(2) $$\frac{1}{18}\sum_{i=1}^{3}\sum_{j=1}^{3}\;\frac{\left[3\cos^{2}\left(\theta_{ij}\right)-1\right]}{r_{j}^{3}}$$

where *r*_j_ is the distance between the methyl proton *j* and a distal proton and *θ*_ij_ is the angle between the vectors formed between a distal proton and the two methyl protons *i* and *j*.

## RESULTS AND DISCUSSION

### Mutational variation of the slow exchange conformational equilibrium in FKBP12

**Figure 1:**
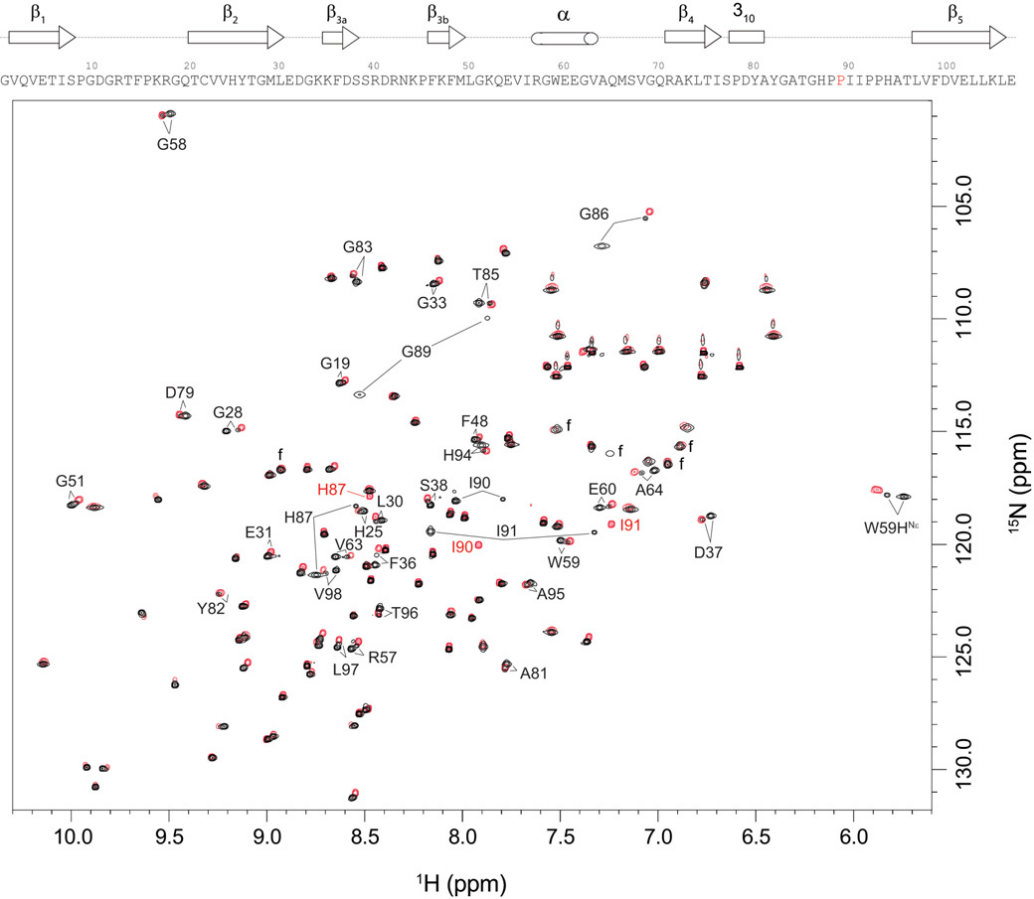
^1^H–^15^N 2D NMR correlation spectra of wild-type and the G89P variant of FKBP12 Cross-peaks from the G89P variant are indicated in red. Resonances that are doubled in the wild-type spectrum are identified. The Ala^84^ resonances were not observed in this spectrum due to severe linebroadening presumably because of the rapid amide hydrogen exchange as observed in FKBP12 [27]. f, folded side chain resonances.

The largest chemical-shift differences for the doubled amide resonances of FKBP12 are seen for residues in the 80′s loop [22]. The resonance doubling extends into the first two residues of the β_5_-strand and the structurally contiguous residues of the adjacent β_2_- and β_3α_-strands (Figure 1). The doubling also extends backwards along the chain into the 3_10_ turn and into the nearby α-helix which bears the Trp^59^ indole side chain that forms the base of the active-site cleft. The residues exhibiting resonance doubling extend beyond the active site to the beginning of the 50′s loop that abuts the C-terminal end of the α-helix.

We reported previously the crystal structure and ^15^N relaxation analysis of an H87V variant of FKBP12. Despite the close similarity of the H87V structure [31] (0.25 Å C^α^ RMSD with respect to the 0.92 Å wild-type structure), the slow resonance doubling transition is completely quenched throughout the protein [22]. Chemical shift-based prediction of the backbone torsion angles for the minor slow exchange state yielded a negative ϕ torsion angle for Gly^89^ as compared with a value of +103° reported in the 0.92 Å resolution structure of FKBP12 [31] (PDB code 2PPN).

In the present study, the G89A mutation was introduced so as to shift the conformational equilibrium towards a negative ϕ value for this residue. The 2D ^1^H,^15^N HSQC spectrum of the G89A variant yielded a pattern of amide resonance doublings with chemical-shift values quite similar to that observed for the wild-type protein [22], although the relative intensity of cross-peaks from the two slow exchange conformational states is markedly different (Supplementary Figure S1 at http://www.biochemj.org/bj/458/bj4580525add.htm). In the wild-type spectrum the minor slow exchange state represented only 12% of the total population, whereas in the G89A variant the analogous set of cross-peaks yielded 67% of the protein signal. This change in population corresponded to a 6.7 kJ/mol difference in the relative stability of the two conformations that was induced by the alanine residue substitution.

To examine further how enforcing a negative ϕ torsion angle at residue 89 affects the structure and dynamics of FKBP12, the G89P variant was analysed by NMR. The proline residue substitution eliminates peak doubling for all of the amide resonances (Figure 1). The similarity in chemical-shift behaviour for the G89P variant and the minor slow exchange conformation of the wild-type protein indicated strongly a corresponding similarity in structure. The only marked differences in the superimposition of these two sets of resonances occurred at residue 89, for which the G89P variant lacked an amide resonance, and Ile^90^ for which the ^15^N of the G89P was shifted downfield as anticipated from the inductive effects resulting from the side chain substitution for the preceding residue [42,43].

### Crystal structure for a G89P variant of FKBP12 at 1.50 Å resolution

**Figure 2:**
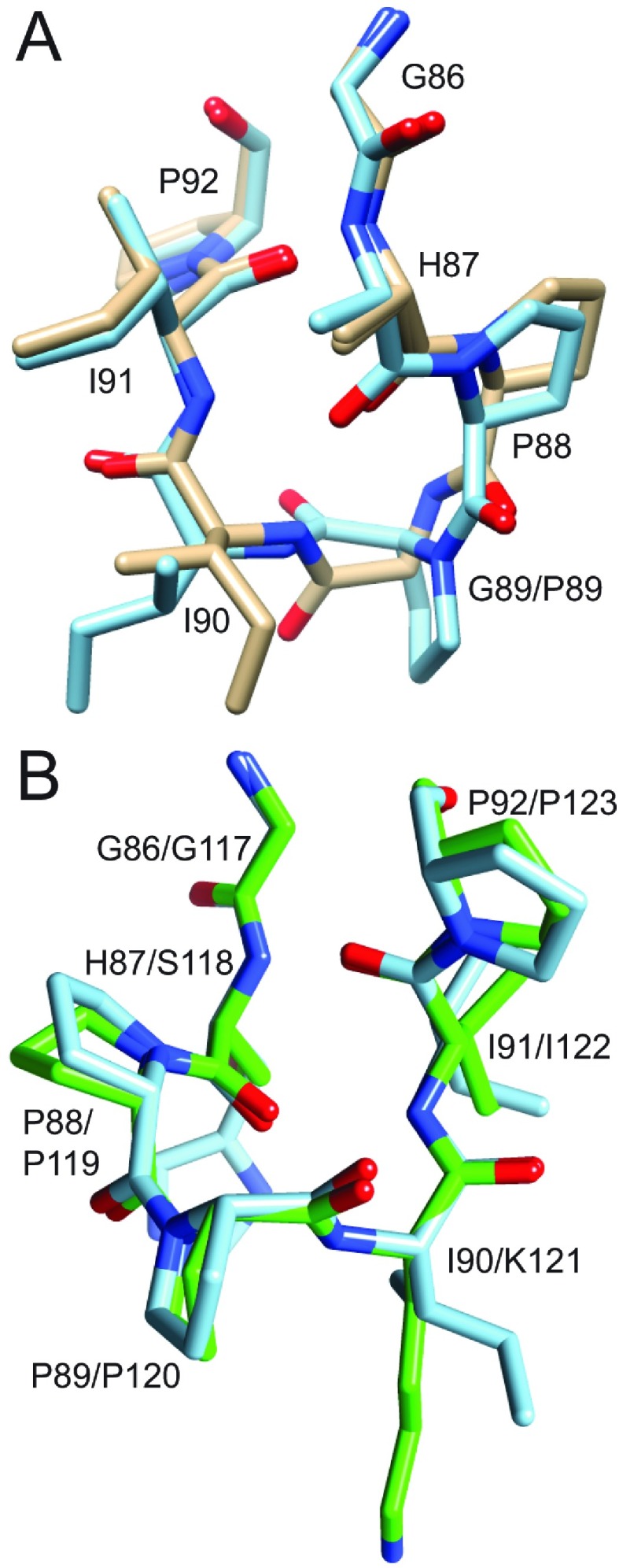
Superimposition of region surrounding the Pro^88^–Pro^89^ peptide bond in the crystal structure of the G89P variant (blue) as compared with the wild-type FKBP12 (gold) and to the homologous segment from the first FKBP domain of FKBP52 (green) (**A**) In the comparison between the two FKBP12 structures, the His^87^ side chain is truncated at C^β^ to facilitate visualization of the backbone conformations. The *cis*-peptide linkage in the G89P structure perturbs the backbone of residues 88–90, whereas the preceding and following residues superimpose fairly closely. (**B**) As viewed from the opposite face of the loop, the structural changes induced by the *cis*-peptide linkage in the G89P variant yields a backbone conformation that closely follows that of the first FKBP domain of FKBP52 (PDB code 1N1A).

Taking advantage of the close structural similarity between the wild-type and the evolutionarily conservative C22V variant of FKBP12 and the increased stability of the cysteine residue-free variant [22], the G89P mutation was introduced into the C22V background for crystallization trials. Crystals were grown in the *C*2 space group using microseeding from crystals of the C22V,H87V variant [22]. Diffraction data were collected to 1.50 Å resolution using an in-house diffractometer. Following refinement, the backbone ϕ and ψ torsion angles for Pro^89^ were found to be (−92 and −12) with the ϕ value being nearly 180° from that of Gly^89^ in the wild-type structure. Furthermore, this crystal structure adopted a *cis*-peptide conformation for the linkage between Pro^88^ and Pro^89^. Superimposition with the high-resolution wild-type structure indicated that, although the conformation of residues 89 and 90 differed markedly, the residues preceding Pro^88^ and those following Ile^90^ aligned closely (Figure 2A).

The sequence at the tip of the 80′s loop differed by only one residue between the first FKBP domain of FKBP52 (PPKIPP) and the G89P variant of FKBP12 (PPIIPP). A *cis*-peptide linkage at the position homologous with Pro^89^ has been reported in crystal structures of this FKBP52 domain (e.g. PDB codes 1N1A [44] and 4LAV [45]). This region of the 80′s loop in the G89P structure superimposed closely with that of the PDB code 1N1A structure for FKBP52 (Figure 2B).

If the minor slow exchange state of FKBP12 corresponds to the transition of the Gly^89^ peptide linkage to a *cis* configuration, the crystal structure of the G89P variant provides a ready explanation for how the H87V substitution for FKBP12 might suppress this transition. In the crystal structure of the H87V variant, the C^γ2^ atom of that valine residue is 3.7 Å from the C^δ1^ atom of the evolutionarily invariant Tyr^82^ side chain [22]. When a valine residue side chain was modelled on to His^87^ of the G89P crystal structure, the separation between the valine C^γ2^ atom and the C^δ1^ atom of Tyr^82^ was reduced to 2.9 Å, suggestive of a substantial steric hindrance to such a conformational transition.

### Glycine *cis*-peptide configuration at residue 89 in wild-type FKBP12

If the peptide linkage of Gly^89^ in wild-type FKBP12 assumes a *cis*-peptide linkage in the minor state of the slow exchange transition, this implies that this critical protein-recognition loop of FKBP12 adopts a FKBP52-like conformation in a substantial fraction of the conformational distribution. One indirect line of support for this possibility is the 70 kJ/mol activation energy that was determined for the slow exchange process in FKBP12 [22], which is typical for *cis*–*trans* isomerization of peptides. On the other hand, non-proline residue *cis*-peptide linkages are quite rare in protein crystal structures, occurring at a frequency of only 0.03% [46]. To demonstrate directly whether the minor slow exchange state of FKBP12 adopts a *cis*-peptide linkage at this residue, 3D ^13^C-^13^C-^1^H NOESY measurements were carried out to determine the presence of a short range distance between the H^α^ atoms of Pro^88^ and Gly^89^ in the minor conformation. For the more common *trans*-peptide linkage, the distance between sequential H^α^ atoms is ~4.5 Å, nearly independent of the intervening ψ_i_ and ϕ*_i_*_+1_ torsion angles [47]. On the other hand, assuming that the minor slow exchange form of the wild-type protein adopts the geometry of this linkage, as found in the G89P crystal structure, the distances from the Pro^88^ H^α^ to the two Gly^89^ H^α^ atoms were 2.0 and 3.4 Å.

**Figure 3:**
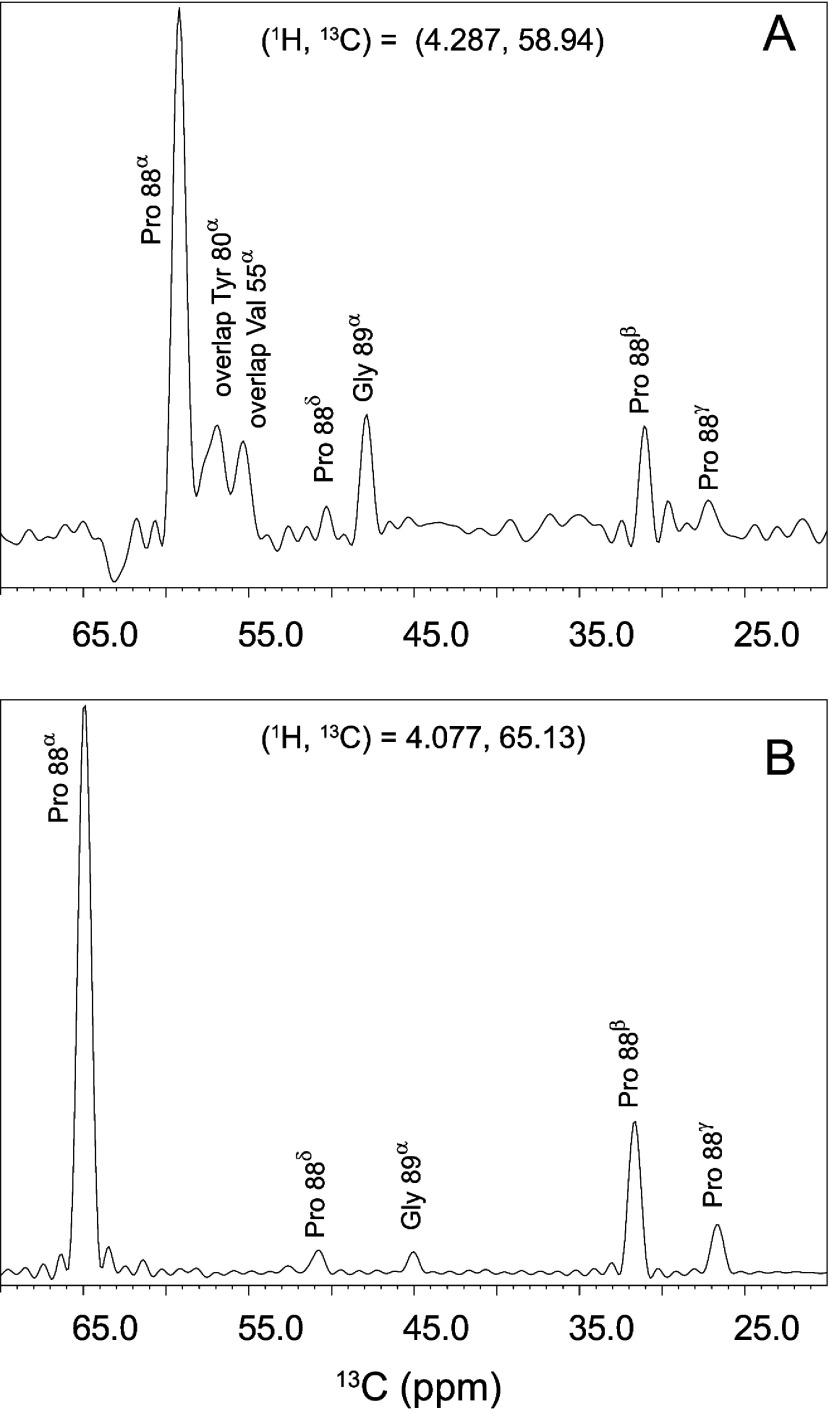
1D slices from the 3D ^13^C-^13^C-^1^H NOESY spectrum of wild-type FKBP12 (**A**) At the ^1^H and ^13^C frequencies for the C^α^ of Pro^88^ in the minor slow exchange state the NOE cross-peak correlating the sequential C^α^ of Gly^89^ is more intense than the corresponding NOE cross-peak correlating the intraresidue Pro^88^ C^β^ interaction. (**B**) In contrast, the corresponding sequential NOE connectivity correlating the C^α^ nuclei of Pro^88^ and Gly^89^ in the major slow exchange state is comparatively far less intense than the intraresidue NOE to the Pro^88^ C^β^.

For the minor state resonances of wild-type FKBP12, the intensity of the sequential NOE transfer from the ^1^H^α^ resonances of Gly^89^ to the ^1^H^α^ of Pro^88^ was more intense than the intraresidue NOE transfer to Pro^88 1^H^α^ from its own ^1^H^β^ resonances (Figure 3A). In contrast, for the major slow exchange state the sequential NOE transfer from the ^1^H^α^ resonances of Gly^89^ to the ^1^H^α^ of Pro^88^ was much weaker than the intraresidue transfer from the ^1^H^β^ resonances (Figure 3B). A similar pattern of NOE cross-peak intensities were observed for transfer in the reverse direction from the ^1^H^α^ of Pro^88^ to the ^1^H^α^ resonances of Gly^89^ for both the major and minor slow exchange states.

### NMR chemical-shift analysis of the backbone conformations for the 80′s loop

Having established that the extensive resonance doubling of FKBP12 arises from the slow *cis*–*trans* isomerization of the peptide linkage between Pro^88^ and Gly^89^, we then examined the structural basis for the much more rapid linebroadening conformational dynamics of the 80′s loop. The transition from the wild-type FKBP12 crystal structure to a conformation resembling the G89P crystal involved not only the change to the Gly^89^ *cis*-peptide configuration. The ϕ torsion angle of Gly^89^ is +103° in the high-resolution crystal structure of wild-type FKBP12 [31], whereas the five-membered ring of the homologous Pro^120^ in the FKBP52 structures forces the ϕ angle to be negative. To examine the plausibility that Gly^89^ of FKBP12 might undergo a relatively rapid transition between positive and negative ϕ angle values while remaining in the predominant *trans*-peptide configuration, the other crystal structures of FKBP52 were considered. As noted above, two crystal structures of FKBP52 (PDB codes 1N1A [44] and 4LAV [45]) exhibit a *cis*-peptide linkage between Pro^119^ and Pro^120^. However, there are two other crystal structures of FKBP52 (PDB codes 1Q1C [48] and 4LAW [45]) for which this peptide linkage is in a *trans* configuration. These latter two crystal structures of FKBP52 can provide models for the conformation of the 80′s loop of FKBP12 in which Gly^89^ switches to an ϕ angle<0, whereas its peptide linkage remains in a *trans* configuration.

*T*_2_ transverse relaxation experiments serve to measure the ^15^N linebroadening effects which arise from the difference in chemical shift for a given nucleus that interchanges between conformational states in the microsecond to millisecond timeframe. A number of empirical algorithms have been developed for predicting NMR chemical shifts from protein structures [49–52]. One widely used algorithm is the SPARTA+ program of Shen and Bax [49] which has applied an artificial neural network analysis against a database of 580 proteins to derive its parameterization. Consistent with the results from other such chemical-shift prediction algorithms, SPARTA+ yielded an S.D. of 2.45 p.p.m. in its predictions of backbone ^15^N chemical shifts for a set of 11 validation proteins, an appreciably lower precision than was obtained for the ^13^C′, ^13^C^α^, ^13^C^β^, ^1^H^α^ and ^1^H^N^ chemical shifts. In modelling the linebroadening for the 80′s loop resonances of FKBP12, the ^15^N chemical shift predictions were only applied in a differential mode in anticipation of an increased precision in the resultant analysis.

**Figure 4:**
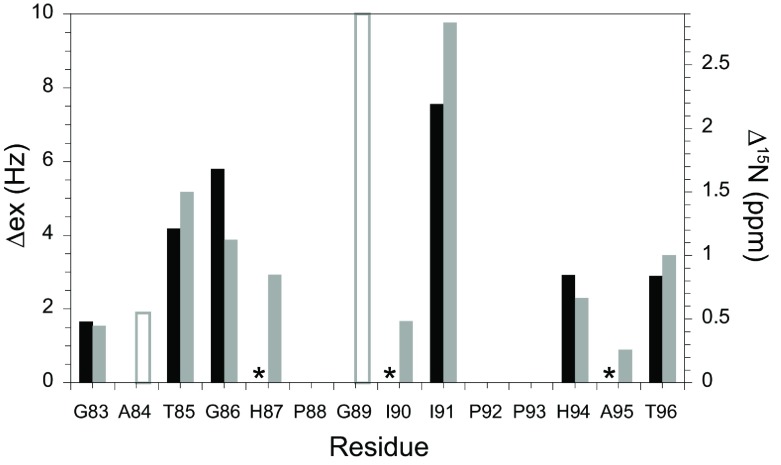
Correlation between the ^15^N conformational exchange linebroadening (Δex) in the 80′s loop of FKBP12 (black) and the differential chemical-shift predictions derived from two crystal forms of FKBP52 (grey) Model-free [41] analysis of the R_1_, R_2_ and NOE ^15^N relaxation measurements was used to estimate conformational exchange linebroadening [22]. The differences in ^15^N chemical shift were predicted with SPARTA+ [49] applied to FKBP12 (PDB code 2PPN [31]) and the first FKBP domain of FKBP52 (PDB code 1Q1C [48] and non-equivalent monomers of PDB code 4LAW [45]). An asterisk indicates not statistical significant conformational exchange linebroadening (<~0.4 Hz). The amide resonances for both Ala^84^ and Gly^89^ are severely broadened due to rapid hydrogen exchange [22], precluding a reliable determination of conformational exchange linebroadening, and the predicted chemical shift differences for these two residues are indicated as open bars.

Exploiting the fact that these chemical shift predictions are dominated generally by the local backbone geometry, the crystal structures for the 80′s loop in FKBP12 [31] (PDB code 2PPN) and the first FKBP domains of FKBP52 (PDB codes 1Q1C [48] and 4LAW molecules A and B [45]) were transformed to a common sequence by converting the disparate residues into alanine, except for the site of the *cis*-peptide transition for which the FKBP52 structures were converted into Gly^120^. The predicted ^15^N chemical-shift values from the FKBP12-derived structure were compared against those from the three FKBP52-derived structures, both individually and as a simple average over those three structures. In comparing these predicted differences in ^15^N chemical shift with the experimentally observed ^15^N linebroadening effects [22], averaging the predictions over the three FKBP52-derived structures provided the strongest correlation (Figure 4).

Given the substantial challenges for quantitative structure-based predictions of conformational exchange linebroadening effects, the degree of correlation between the predicted and observed linebroadening in the 80′s loop of FKBP12 suggests strongly that Gly^89^ transiently adopts a negative ϕ torsion angle when that residue is in a *trans*-peptide configuration. On the other hand, the absence of ^15^N conformational exchange linebroadening for the 80′s loop when Gly^89^ is in the *cis*-peptide configuration of the minor slow exchange state [22] suggests that positive ϕ torsion angles for Gly^89^ are not energetically accessible from that state in a comparable population or timeframe to that observed for the *trans*-peptide configuration. The simplest mechanistic interpretation consistent with these data is that the two slowly interchanging Gly^89^ peptide configuration states, monitored by the extensive resonance doubling, interchange with each other only via a more weakly populated transient state in which the ϕ torsion angle of Gly^89^ is negative, whereas the peptide configuration remains *trans*.

As noted above, the H87V substitution suppresses all of the amide resonance doubling, presumably by its steric collision with the Tyr^82^ ring in the Gly^89^ *cis*-peptide configuration. This substitution also suppresses the microsecond to millisecond linebroadening transition for the resonances in the 80′s loop [22]. We propose that this linebroadening arises from a ~180° shift in the ϕ torsion angle of Gly^89^. The ψ angle of Pro^88/119^ also shifted by an average of 130° between the wild-type FKBP12 and three FKBP52 crystal structures in the *trans*-peptide configuration. These three crystal structures of FKBP52 can be used to examine this model for the quenching of linebroadening in the 80′s loop. Residue 118 (homologous with His^87^ of FKBP12) was computationally transformed into a valine for each of these three FKBP52 structures. For PDB code 1Q1C and monomer A of PDB code 4LAW, this valine residue side chain must strongly overlap with either the aromatic ring of Tyr^113^ (homologous with the evolutionarily invariant Tyr^82^) or with the backbone of Ile^122^. Such a steric conflict could be expected to preclude the transition of Gly^89^ to a negative ϕ torsion angle in the H87V variant, thus eliminating the linebroadening effect for the 80′s loop. A caveat to this detailed structural interpretation is that in the significantly differing local geometry of monomer B in the PDB code 4LAW structure of FKBP52 the introduction of the valine residue side chain at residue 118 does not appear to yield a severe steric conflict.

Both the H87V [53] and G89P [54] variants have been shown to have an equivalent peptide isomerase activity with that of wild-type FKBP12 in the standard assay using blocked tetrapeptides. The H87V variant appeared to block the resonance doubling and the conformational linebroadening of the 80′s loop by constraining Gly^89^ to remain in the *trans*-peptide configuration with a positive ϕ torsion angle. The G89P variant appeared to block the resonance doubling and the conformational linebroadening of the 80′s loop by constraining residue 89 to remain in the *cis*-peptide configuration with a negative ϕ torsion angle. The linebroadening transition and the much slower resonance doubling transition did not appear to participate in the peptide isomerization activity of this enzyme, at least for simple conformationally unstructured peptides. Any functional relevance of this conformational plasticity/dynamics may instead relate to a role in the protein-recognition interactions of FKBP12.

### Perpendicular reorientation of the Trp^59^ indole at the base of the active-site cleft in a G89P variant of FKBP12

**Figure 5:**
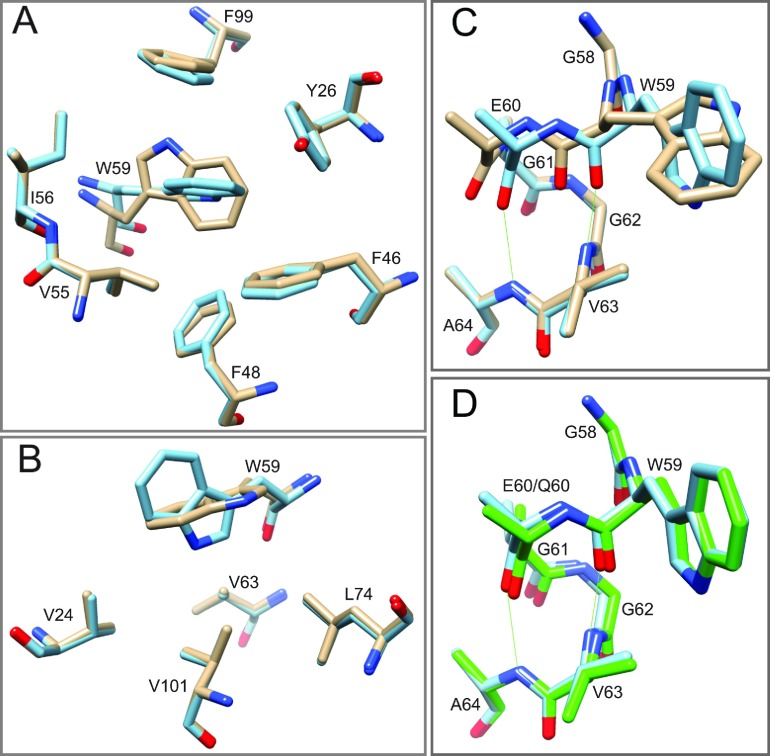
Superimposition of the active-site region surrounding Trp^59^ and Glu/Gln^60^ from the wild-type (gold), G89P (blue) and E60Q (green) crystal structures (**A**) Above the plane of the indole ring in the PDB code 2PPN structure, the aromatic rings of Tyr^26^, Phe^46^, Phe^48^ and Phe^99^ form much of the wall of the active-site cleft. (**B**) Underneath the indole ring of Trp^59^, the C^δ1^ and N^ε1^ of that residue can occupy a cavity formed by the side chains of Val^24^, Val^63^, Leu^74^ and Val^101^ which is unperturbed by this structural rearrangement. (**C**) The superimposition of the wild-type and G89P crystal structures illustrates a displacement of the backbone such that in the G89P structure canonical geometries are established for the hydrogen bonds between Glu^60^ O and Ala^64^ H^N^ and between Trp^59^ O and Val ^63^ H^N^. (**D**) This region of the crystal structure of the E60Q variant is highly similar to that for G89P.

The most unexpected aspect of the G89P crystal structure was that the indole ring of Trp^59^ at the base of the active-site cleft was oriented perpendicular to the conformation observed in each of the more than 30 crystal structures of wild-type FKBP12 (Figure 5A). The aromatic rings of Tyr^26^, Phe^46^, Phe^48^ and Phe^99^ and the side chains of Val^55^ and Ile^56^ form the walls of the active-site cleft surrounding the Trp^59^ ring. The reorientation of the indole ring in the G89P crystal structure occluded much of the volume of the active-site cleft, conflicting with the binding geometry observed in the crystal structures of the FK506- and rapamycin-ligated proteins. As compared with the wild-type crystal structure, the aromatic rings of Phe^48^ and Phe^99^ in the G89P structure appeared to move slightly inward towards the reoriented indole ring. The conformation of the residues that lie behind the Trp^59^ indole ring was essentially identical for the wild-type and the G89P structures. A small cavity lies beneath the indole ring in the wild-type structure, and the nearby side chains of Val^24^, Val^63^, Leu^74^ and Val^101^ were essentially unperturbed by the rotation of the indole ring (Figure 5B).

This perpendicular reorientation of the Trp^59^ aromatic ring in the G89P crystal structure was strikingly similar to that reported for the E60Q (PDB code 2PPP) and E60A (PDB code 2PPO) variants of FKBP12 [31]. In addition, the backbone of the helical residues surrounding Trp^59^ in the G89P crystal structure was closely similar to that observed in the E60Q (and E60A) structure (Figure 5D). In contrast, the hydrogen-bonding interactions for Trp^59^ O to Val^63^ H^N^ and Glu^60^ O to Ala^64^ H^N^ were significantly irregular in the crystal structure of wild-type FKBP12 with the carbonyl groups being shifted laterally by over 1 Å with respect to the amide bond vectors as compared with the canonical hydrogen-bonding geometry seen in the G89P structure (Figure 5C). Regarding the role of crystal packing interactions in the present study, it may be noted that the *P*2_1_ crystal forms for the 0.94 Å resolution structure of the E60Q variant and the 1.29 Å resolution structure of the E60A variant were isomorphous with that used in the 0.92 Å resolution structure of wild-type FKBP12 [31]. The G89P variant crystallized in the *C*2 space group.

Saven and colleagues noted that the Glu^60^ side chain in their high-resolution structure of wild-type FKBP12 extends towards the backbone of the 50′s loop to co-ordinate the amide hydrogens of Gly^51^ and Gln^53^, as well as to a highly conserved solvent-inaccessible water molecule [31]. Introduction of glutamine at position 60 of FKBP12 results in the flipping of the peptide unit that links Lys^52^ and Gln^53^ and a shift in co-ordination for the buried water molecule. Although the backbone of the 50′s loop in the E60A variant remains similar to that of the wild-type protein, the side chain interaction of residue 60 with the buried water molecule is obviously disrupted. The authors [31] argued that these altered interactions of residue 60 with the buried water molecule results in a shift in the α-helix, which, in turn, presses the indole ring more tightly against the ring of Phe^99^ thus driving the reorientation of the Trp^59^ side chain.

**Figure 6:**
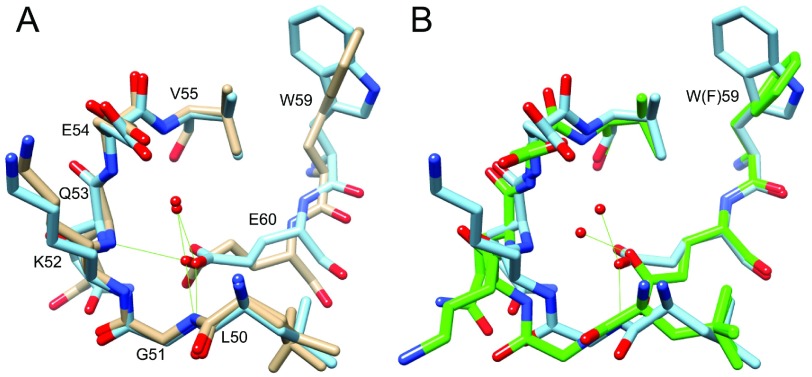
Interactions of Glu^60^ with the 50′s loop in wild-type (gold), G89P (blue) and W59F (green) FKBP12 crystal structures (**A**) The Glu^60^ side chain differs in the G89P structure primarily by adopting an extended *trans* χ_1_ rotamer conformation which enables the shift in the backbone into a canonical α-helical hydrogen-bonding geometry. (**B**) The crystal structure of the W59F variant [55] exhibits a similarly extended *trans* χ_1_ rotamer conformation for Glu^60^ and canonical α-helical hydrogen-bonding geometry, although the phenyl ring of Phe^59^ remains flat across the bottom of the active-site cleft.

The backbone of the 50′s loop and the position of the buried water molecule in the crystal structure of the G89P variant closely superimposed with that of wild-type FKBP12 (Figure 6A). The carboxy O^ε1^ atom of Glu^60^ preserved its hydrogen-bonding interactions with the amide hydrogens of Gly^51^ and Gln^53^ and with the buried water molecule. The similarity in the interactions of the Glu^60^ side chain with the backbone of the 50′s loop and the buried water molecule for the wild-type and G89P structures suggested that disruption of these interactions were sufficient, but not necessary, to shift the backbone of Trp^59^ and Glu^60^ into a canonical α-helical hydrogen-bonding geometry and enable the reorientation of the indole ring.

The most notable difference for Glu^60^ between the crystal structures of wild-type FKBP12 and the G89P variant is the transition of the side chain from a gauche^−^ to a *trans* χ_1_ torsion angle, which allows the backbone atoms of Trp^59^ and Glu^60^ to shift towards the active site indole ring without disrupting the hydrogen-bonding interactions described above. Such a shift from a gauche^−^ to a *trans* χ_1_ torsion angle for the Glu^60^ side chain was reported previously in the crystal structure of the W59F variant of FKBP12 [55]. In this case, the backbone atoms of Phe^59^ and Glu^60^ underwent a similar shift to that observed in the structure of the G89P variant (Figure 6B). On the other hand, the smaller phenyl side chain at residue 59 does not become tightly pressed against the aromatic ring of Phe^99^ when it maintains an orientation along the bottom of the active-site cleft similar to that of the tryptophan ring in the wild-type FKBP12 structure. In comparing the crystal structures of the W59F and G89P variants, it appeared that disruption of the steric interaction between the indole ring of Trp^59^ and the phenyl ring of Phe^99^ was sufficient to enable the backbone of Trp^59^ and Glu^60^ to shift into a canonical α-helical hydrogen-bonding geometry.

Crystal structures have been reported for four of the other six human FKBP domains which contain a tryptophan ring at the base of the active-site cleft [44,56–58]. In each of these four structures, Glu^60^ of FKBP12 is replaced by the shorter aspartate side chain which forms similar interactions with the backbone of the 50′s loop. It is not self-evident how these interactions could be preserved while undergoing the shift in the α-helical backbone to allow for an analogous reorientation of the tryptophan ring. A further indication that the reorientation of the active site indole side chain may be energetically disfavoured in these other FKBP domains is that the unoccupied volume beneath the plane of the Trp^59^ ring in FKBP12 appears to be occluded in these other crystal structures. In the example of FKBP51 [56], the aliphatic side chains underlying the indole ring closely superimpose on those of FKBP12 illustrated in Figure 5(B), except Ile^132^ which was substituted for Val^101^. This substitution resulted in the isoleucine residue C^δ^ atom occupying the position of the void space in the FKBP12 crystal structure. Similarly, Ile^94^ and Ile^105^ of FKBP52 [45] replace the less bulky pair of Val^63^ and Leu^74^ in FKBP12, resulting in a reduced void volume beneath the tryptophan ring. If the transition of the indole ring to the perpendicular orientation is substantially more populated in FKBP12, as compared with these other FKBP domains, such a conformation might prove amenable to selective drug inhibition.

### Reorientation equilibria and dynamics for the active site indole ring in wild-type FKBP12

The structural analysis of these FKBP12 variants support the possibility that there may be only a modest energy difference between the canonical and the perpendicular orientations for the indole ring of Trp^59^ in the wild-type protein. Potential evidence for internal mobility of the single indole ring in FKBP12 comes from the time-resolved fluorescence anisotropy measurements of Silva and Prendergast [59] in which they derived an *S*^2^ order parameter of 0.75 for the uncomplexed protein and an *S*^2^ value of ~1 for the FK506-bound complex. In contrast with the three fastest relaxation components for which the amplitudes and time constants were similar for the free and ligated forms of the protein, the time constant of 5.43 ns for the slowest component of the fluorescence anisotropy decay in the FK506-bound protein was decreased to 4.75 ns for uncomplexed FKBP12. The authors analysed these data assuming that this shift in the time constant arises from unresolved contributions from a local conformational transition of the indole ring and the global molecular tumbling [59], leading to a predicted time constant of 3 ns for the internal motion. In their molecular dynamics analysis on the role of the buried water molecule in modulating the conformational dynamics of FKBP12, Park and Saven [60] reported a simulation of the wild-type protein in which the Trp^59^ indole ring transitioned to a perpendicular orientation after 8 ns in a 12 ns simulation. Although no reverse transition was observed, their simulation suggested the energetic accessibility of such a conformation.

In the 3D ^13^C-^15^N-^1^H NOESY spectrum of U-^13^C,^15^N-labelled FKBP12 we observed substantial cross-peaks from the indole H^Nε1^ of Trp^59^ to both methyl groups for Val^24^ and Val^63^ as well as to the C^γ1^ methyl of Val^101^, despite the fact that these methyls lie ~7 Å from the indole H^Nε1^ in the crystal structure of the wild-type protein. To examine this issue in more detail, we prepared a sample of FKBP12 with ^13^C enrichment for the methyl groups of valine, leucine and isoleucine residues with uniform ^15^N and perdeuteration at all other carbon-bound sites as described previously by Kay and colleagues [29]. For most of the moderately intense cross-peaks of the ^13^C-^15^N-^1^H NOESY spectra for valine, leucine and isoleucine methyl-labelled FKBP12, the NOE build-up curves were approximately linear up to a 200 ms mixing time.

**Figure 7:**
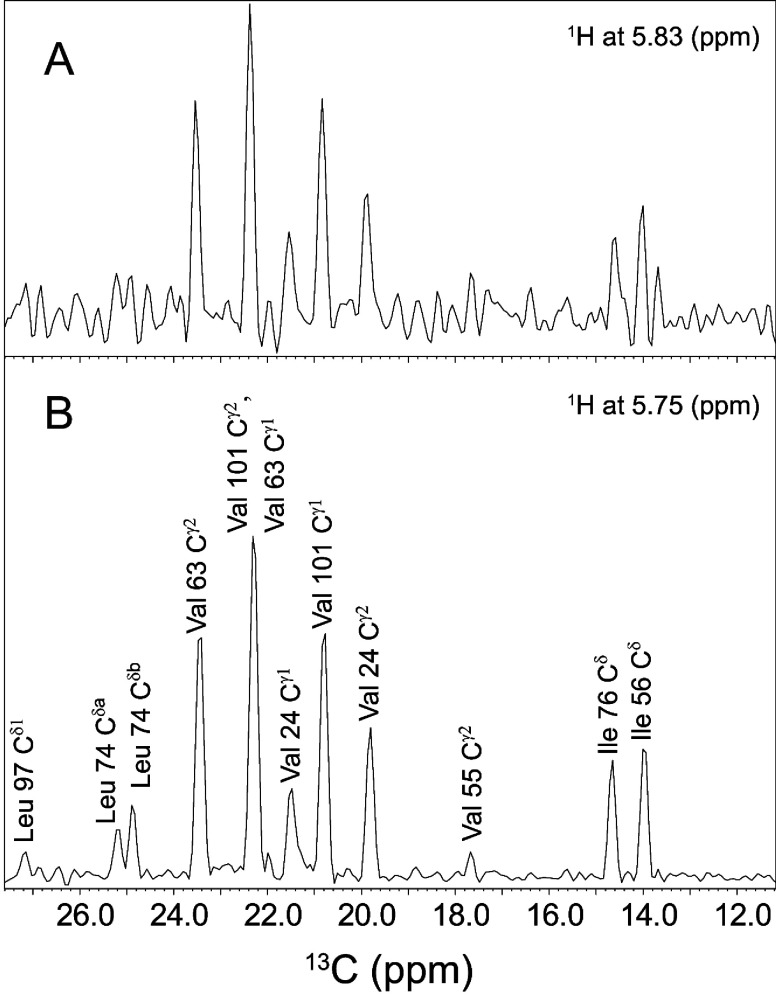
1D slices from the 3D ^13^C-^15^N-^1^H NOESY spectrum at the ^1^H^Nε1^ and ^15^N ^ε1^ (117.75 p.p.m.) frequencies of Trp^59^ NOE cross-peaks with ^13^C-enriched methyl groups in the major (**B**) and minor (**A**) slow exchange states of wild-type FKBP12. The methyl groups of Leu^7^ were not stereochemically assigned.

A 1D ^13^C slice at the ^1^H and ^15^N frequencies of the Trp^59^ indole N–H in the major slow exchange conformational state indicates 11 peaks, most of which correspond to methyl groups that are too distant from the indole H^Nε1^ in the high-resolution structure of wild-type FKBP12 to give rise to the observed intensities (Figure 7B). The analogous 1D slice at the ^1^H and ^15^N frequencies of the Trp^59^ indole N–H in the minor slow exchange conformational state yielded a qualitatively similar pattern (Figure 7A), although the lower sensitivity from this minor state precluded a more detailed analysis.

The methyl–indole H^Nε1^ cross-peak volumes were analysed to test their consistency with FKBP12 adopting a mixture of active-site conformations corresponding to the PDB code 2PPN wild-type and G89P variant crystal structures. The familiar 1/*r*^6^ distance dependence for the NOE cross-relaxation rate typically invokes the assumption of an isolated pair of nuclear spins in the absence of internal motion.

High levels of deuteration suppress strongly spin diffusion effects thus providing a useful approximation of the isolated spin pair assumption. Furthermore, if the rapid internal motion for both of the interacting ^1^H nuclei is isotropic and uncorrelated (similar to the standard assumption of crystallographic *B*-factor analysis for all but the highest-resolution protein structures), the resultant NOE cross-relaxation rate equals that predicted for the two nuclei being fixed at their mean relative positions [61]. Although this exact cancellation of the radial and angular dispersion effects provides a useful approximation for many types of proton pairs, the 3-fold rotational jumps of methyl protons deviates strongly from spherical isotropy. As a result, NOE calculations based on a simple 1/*r*^6^ summation of distances from the methyl protons to a distal proton site give rise to severely foreshortened apparent distances in protein NOESY analyses. In the present study, methyl–indole H^Nε1^ NOESY cross-peaks were analysed under the assumption of separability of the generalized order parameter characterizing the interaction (eqn 2) and this analysis was compared with the results derived from the more commonly used approximations of measuring NOE distances from a unified methyl group centred at either the methyl carbon or the mean position of the methyl protons.

Table 1 lists the normalized experimental volumes for the methyl–indole H^Nε1^ NOESY cross-peaks shown in Figure 7(B). In addition to normalization against the most intense NOE resonance, the volumes for these NOE cross-peaks were normalized against the peak volumes for the corresponding methyl resonances from the 2D ^1^H–^13^C HSQC spectrum to compensate for differences in relaxation behaviour and in the levels of ^13^C enrichment due to incomplete biosynthetic incorporation from the labelled α-oxobutyrate and α-oxoisovalerate during protein expression. Also listed are the distances from each methyl carbon to the indole H^Nε1^ in the PDB code 2PPN wild-type and the G89P variant crystal structures. Distances beyond 7 Å were not included in the calculations and neither were the NOE cross-peaks to the Leu^74^ methyls due to ambiguity in their stereochemical assignment. This residue offers minimal utility in the NOE analysis as the distance from the closest Leu^74^ methyl to the Trp^59^ H^Nε1^ is nearly the same for both the wild-type and G89P crystal structures.

**Table 1:** Generalized order parameter NOE analysis of Trp^59^ indole reorientation in FKBP12 Interactions for methyl–H^Nε1^ distances >7 Å were not included. The ratio of volumes between the normalized NOEs for PDB code 2PPN wild-type/G89P was 0.28. Distance, distance between methyl carbon to the indole H^Nε1^ in the PDB code 2PPN wild-type and the G89P variant crystal structures. NOE(exp), normalized experimental volumes for the methyl–indole H^Nε1^ NOESY cross-peaks.

Residue	Atom	NOE(exp)	Distance_2PPN_ (Å)	NOE (*S*^2^)	Distance_G89P_ (Å)	NOE (*S*^2^)
Ile^56^	C^δ1^	0.795	4.38	0.903		
Ile^76^	C^δ1^	0.828	4.14	1.000		
Val^55^	C^γ2^	0.048			6.59	0.018
Val^24^	C^γ2^	0.460			3.89	0.661
Val^101^	C^γ1^	0.650	6.82		3.37	0.858
Val^24^	C^γ1^	0.308			4.54	0.173
Val^101^*	C^γ2^	1.000	4.98	0.348	3.32	1.000
Val^63^	C^γ2^	0.651	6.88	0.054	3.60	0.613
Leu^97^	C^δ1^	0.050	6.27	0.092		

*This cross-peak also includes the degenerate Val^63^ C^γ1^ which contributes only 4% of the predicted volume.

**Figure 8:**
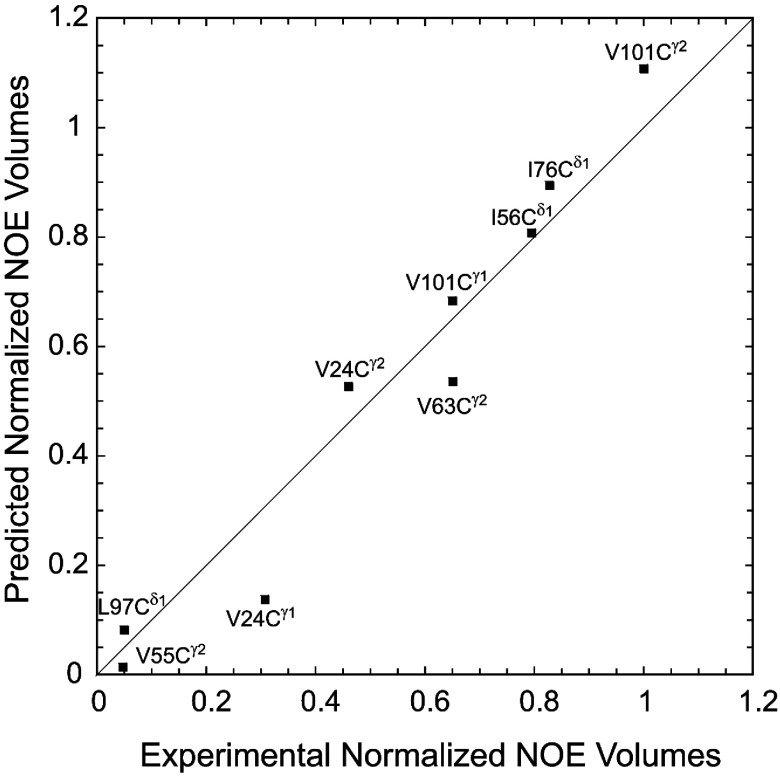
Predicted against observed NOE volumes for the methyl cross-peaks to Trp^59^ H^Nε1^ The experimental NOE volumes were normalized to the maximum value (Val^101^ C^γ2^) and to the volumes of the corresponding ^1^H–^13^C cross-peaks in the 2D HSQC spectrum to compensate for relaxation and differential enrichment effects. Generalized order parameter calculations were performed on the crystal structures of the wild-type protein and G89P variants and then weighted at 80% and 20% respectively.

The relative NOE volumes for each of these methyl–indole H^Nε1^ interactions were predicted from generalized order parameter calculations [41] applied to the two crystal structures. For an admixture of 80% and 20% for the PDB code 2PPN wild-type and the G89P conformations respectively, the observed NOE volumes were fitted to an RMSD of 0.086 with a correlation coefficient (*r*) of 0.976 (Figure 8). A 20% shift to an admixture of 84% and 16% predicts an increase in RMSD to 0.121 with an *r* value of 0.957, whereas, similarly, a 75–25% conformational mixture yields an RMSD of 0.108 with an *r* value of 0.956. When the methyl carbon position was used to model the effective methyl–indole H^Nε1^ distance, the optimal fit was obtained for an 82–18% distribution with an RMSD of 0.134 and an *r* value of 0.955 (Supplementary Figure S2 and Supplementary Table S1 at http://www.biochemj.org/bj/458/bj4580525add.htm). Use of the mean methyl proton position yields an optimum distribution at 79% and 21% with an RMSD of 0.183 and an *r* value of 0.905 (Supplementary Figure S3 and Supplementary Table S2 at http://www.biochemj.org/bj/458/bj4580525add.htm). The markedly worse performance for the use of the mean methyl proton position reflects principally the fact that distal protons that lie along the methyl rotation axis yield elevated predictions for both the radial and angular components of the NOE interaction.

These results indicate significant conformational plasticity in the active site of FKBP12. As there is no apparent linebroadening for the resonances involved, the indole reorientation occurs presumably more rapidly than for the microsecond to millisecond conformational exchange regime. Indication of a much more rapid transition is drawn from the ^15^N relaxation analysis that we reported recently for the backbone amides of FKBP12 [22]. The *R*_1_ and *R*_2_ relaxation rates of 1.50 s^−1^ and 5.56 s^−1^ and the ^15^N NOE value of 0.634 at 14.1 T (600 MHz ^1^H) that we observed for the Trp^59^ N^ε1^ resonance are all well below the values observed for the amides in the well-ordered regions of the backbone (molecular tumbling time τ_c_ of 5.8 ns). Applying the extended model-free analysis [62] to the Trp^59^ N^ε1^ relaxation yielded an overall *S*^2^ order parameter value of 0.63 and a time constant τ_e_ of 1.5 ns for the slow component of internal motion.

For a simple jump model S^2^ can be calculated by eqn (3) [63,64]:

(3) $$S^{2}=1-3p_{a}p_{b}sin^{2}\theta_{\mathrm{ab}}$$

where *p*_a_ and *p*_b_ are the state populations and *θ* is the angle between the indole N–H bond vector orientations which is 83° for the PDB code 2PPN wild-type and G89P structures. A 20% population for the reoriented indole conformation yields an estimated *S*^2^ order parameter of 0.53. Both of the experimental and model *S*^2^ values should be regarded as approximate. In contrast with the fluorescence study cited above [59], the large number of ^1^H–^15^N bond vectors monitored in the NMR relaxation experiments facilitate the deconvolution of contributions from internal and global motion. Nevertheless, the derived order parameter values are still generally less accurate for internal motions having time constants similar to that for the global tumbling of the protein. The simple jump model does not account for the smaller scale librational motions of the ^1^H–^15^N vector which give rise to order parameter values near 0.85 for the well-ordered backbone amides and presumably for the Trp^59^ indole ring as well. Despite these caveats, the experimental evidence for significant conformational dynamics of the tryptophan residue indole ring in the nanosecond timeframe appears to be consistent with the ring reorientation process characterized by the NOE analysis.

### Dual conformers for the active site indole ring in a G89P variant of FKBP12 at 1.20 Å resolution

**Figure 9:**
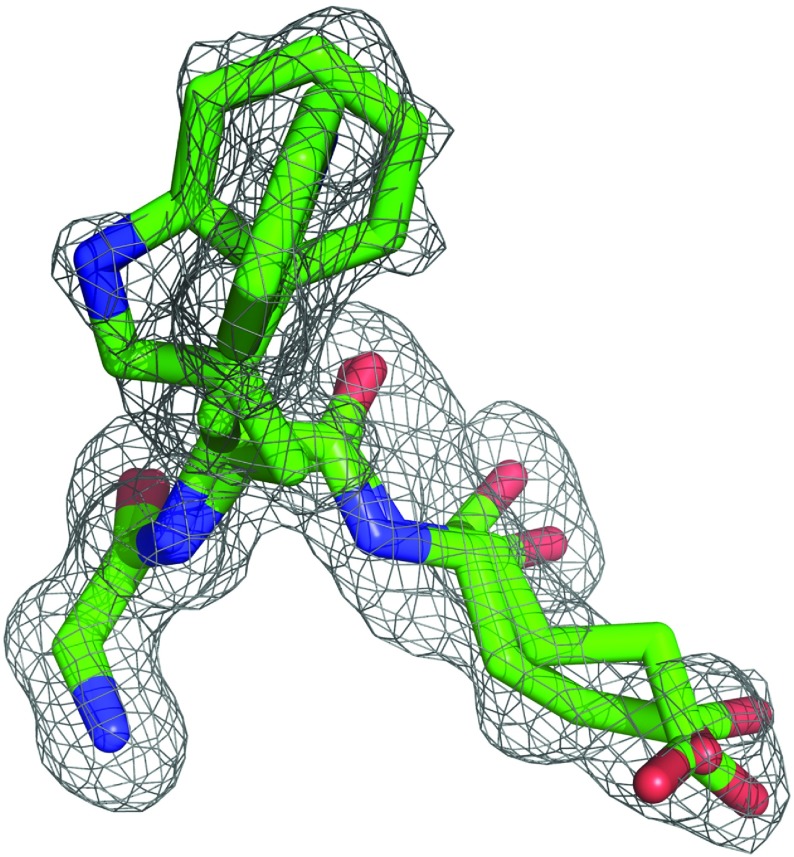
Electron density omit map for Gly^58^, Trp^59^ and Glu^60^ in the active site of the G89P variant of FKBP12 at 1.20 Å resolution Viewed along the plane of the indole ring for the major conformer (occupancy of 0.71), electron density for the perpendicular orientation of the minor conformer is apparent readily. The carbonyl oxygen in the minor conformer of Glu^60^ (occupancy of 0.34) is shifted away from the canonical α-helical hydrogen-bonding geometry seen in the major conformer and to a position close to that observed in the wild-type FKBP12 structure [31]. The contour level for the electron density grid is 0.1261 *e*/Å^3^=2*σ*.

Given the small free-energy difference between the two perpendicular orientations that the active site indole ring of wild-type FKBP12 occupies in solution and the modest differences surrounding that ring in the wild-type and G89P variant crystal structures, we examined further whether the crystal structure of the G89P variant might also yield evidence for conformational heterogeneity at this site. Since the crystals for this variant diffracted well beyond the 1.50 Å resolution data that we had initially collected on our in-house diffractometer, we collected a second dataset to 1.20 Å resolution at the National Synchrotron Light Source (Table 2). During the later stages of refinement, dual conformers were allowed for both Trp^59^ and Glu^60^. Minor conformers for the two residues were obtained at occupancies of 0.29 and 0.34 respectively. When viewed edge-on for the major conformer of Trp^59^, the electron density for a perpendicular orientation of the indole ring is readily apparent (Figure 9). In the minor conformer of Glu^60^, the main chain carbonyl oxygen was shifted towards the 50′s loop into the position seen in the wild-type FKBP12 crystal structure. Although the Glu^60^ C^γ^ of the minor conformer was not well fitted to the electron density, the derived side chain χ_1_ torsion angle was shifted towards the orientation seen in the wild-type FKBP12 structure.

**Table 2:** Crystallographic data collection, refinement and model details (PDB code 4N19) Values in paretheses are for the highest-resolution shell.

Parameter	Value
Data collection	
Resolution range (Å)	35–1.20 (1.3–1.20)
Number of unique reflections	23973
Redundancy	5.7 (2.2)
Completeness (%)	72.7 (8.5)
Average *I/σ(I)*	48.5 (2.5)
*R*_merge_ (%)	6.0 (34)
Refinement	
Resolution limits (Å)	35–1.20
Number of reflections	23973
*R*_work_ (%)	12.6
*R*_free_ (%)	18.0
Non-hydrogen atoms	
Protein	891
SO_4_^2−^	1
Water	148
Average *B* (Å^2^)	
All atoms	20
Solvent	41
Geometry	
RMSD bond length (Å)	0.02
RMSD bond angle (°)	2.28

The two perpendicular orientations of the Trp^59^ indole ring exhibited by the 1.20 Å resolution structure of the G89P variant reinforces the observation of a small difference in free energy for those two conformations in solution. The similar populations for the minor conformers of Trp^59^ and Glu^60^ in this crystal structure is consistent with a concerted shift for both the indole ring orientation and the backbone hydrogen-bonding geometry for residues 59 and 60, which, in turn, appears to be at least partially coupled to a reorientation of the Glu^60^ side chain.

## Online data

Supplementary data
