# Supplementary material for: Structural basis of conformational transitions in the active site and 80′s loop in the FK506-binding protein FKBP12
Source: Biochem J. 2014 Feb 28;458(Pt 3):525–36. doi: 10.1042/BJ20131429 (PMC3940039; doi:10.1042/BJ20131429)
Supplement: Supplementary data [file bj4580525add.pdf]

Biochem. J. (2014) **458**, 525–536 (Printed in Great Britain) doi:10.1042/BJ20131429

SUPPLEMENTARY ONLINE DATA

Structural basis of conformational transitions in the active site and 80′s loop in the FK506-binding protein FKBP12

Sourajit M. MUSTAFI*, Matthew BRECHER*, Jing ZHANG*, Hongmin LI*†, David M. LEMASTER*† and Griselda HERNÁNDEZ*†[1]

*Wadsworth Center, New York State Department of Health Empire State Plaza, Albany, NY 12201, U.S.A.
†Department of Biomedical Sciences, School of Public Health, University at Albany (SUNY), Empire State Plaza, Albany, NY 12201, U.S.A.

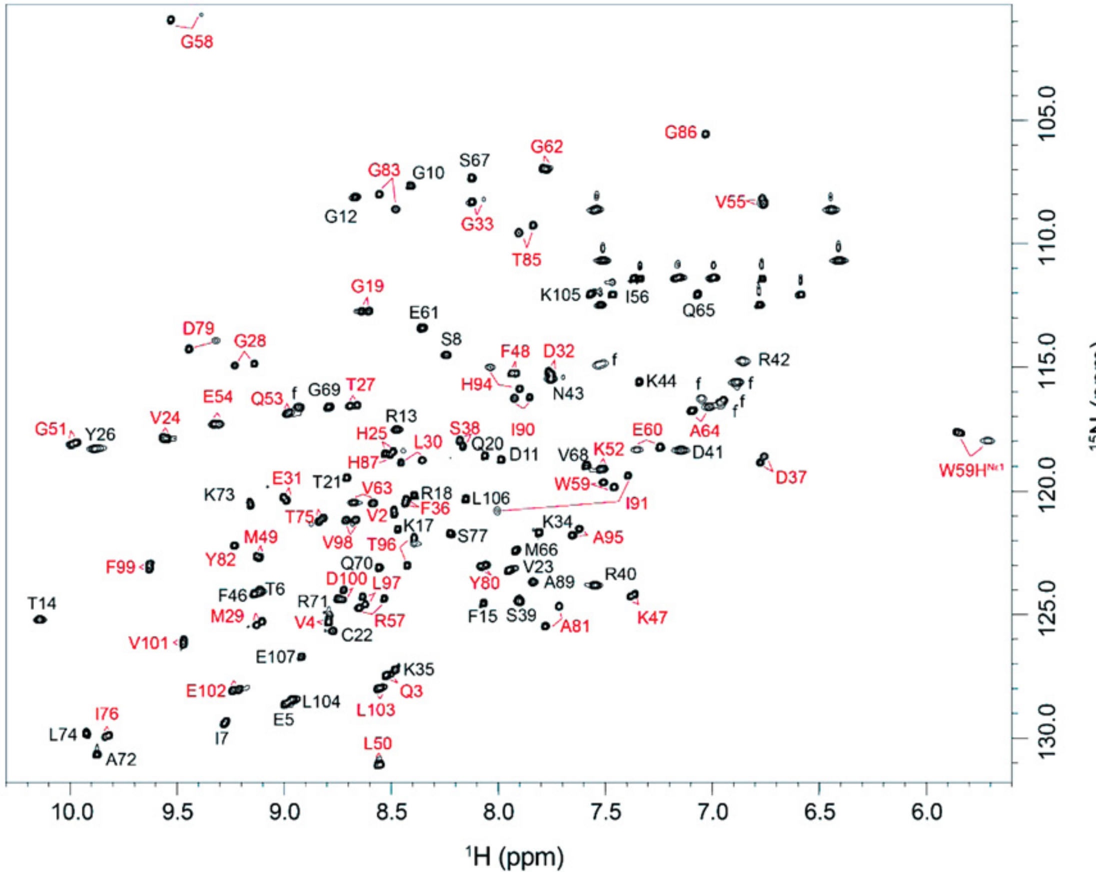

Figure S1 **^{1}H–^{15}N 2D NMR correlation spectrum of the ^{15}N-enriched G89A variant of FKBP12**

Residues exhibiting resolved resonances for the minor slow exchange conformation are indicated in red. The Ala^{84} resonance was not observed in this spectrum due to severe linebroadening presumably because of the rapid amide hydrogen exchange as observed in FKBP12 [1]. f , folded side chain resonances.

[1] To whom correspondence should be addressed (email gch02@health.state.ny.us).
Co-ordinates of the reported protein structure has been deposited in the PDB under code 4N19.

© 2014 The Author(s)
The author(s) has paid for this article to be freely available under the terms of the Creative Commons Attribution Licence (CC-BY) (http://creativecommons.org/licenses/by/3.0/) which permits unrestricted use, distribution and reproduction in any medium, provided the original work is properly cited.

Biochemical Journal www.biochemj.org

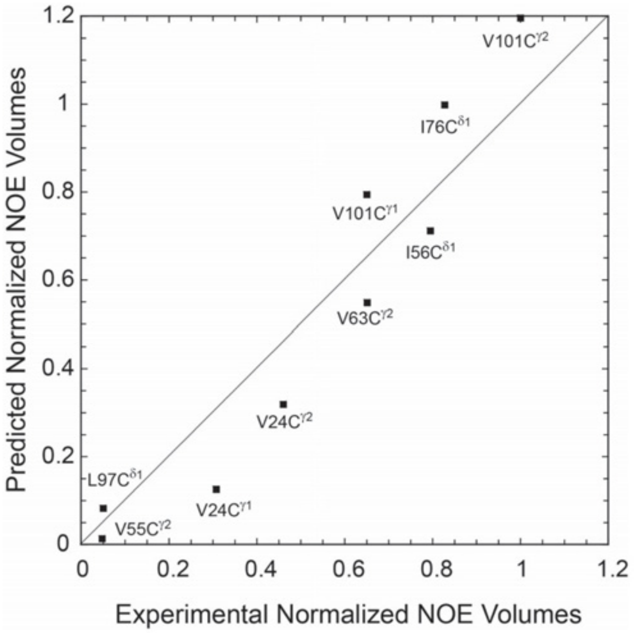

Figure S2 Predicted against observed NOE volumes for the methyl cross-peaks to Trp[59] $H^{N\epsilon 1}$

The experimental NOE volumes were normalized to the maximum value (Val[101] $C^{\gamma 2}$) and to the volumes of the corresponding ^{1}H–^{13}C cross-peaks in the 2D HSQC spectrum to compensate for relaxation and differential enrichment effects. The methyl carbon positions were used to derive NOE volume predictions from the crystal structures of the wild-type protein and G89P variants and then weighted at 82 % and 18 % respectively.

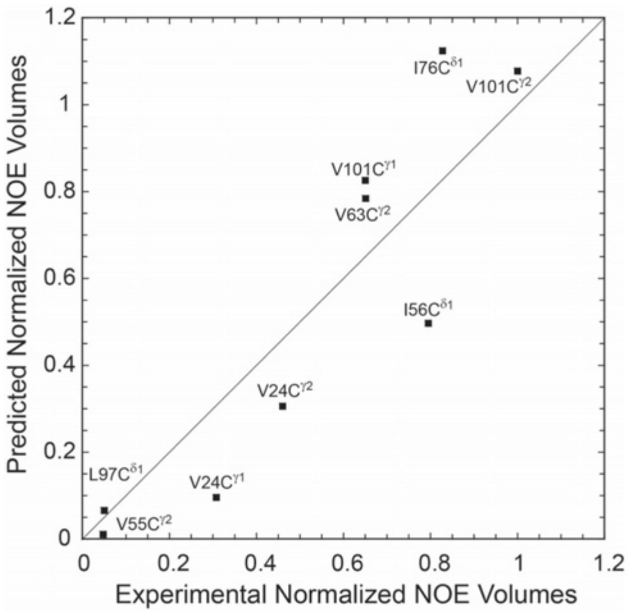

Figure S3 Predicted against observed NOE volumes for the centeroids of methyl cross-peaks to Trp[59] $H^{N\epsilon 1}$

The experimental NOE volumes were normalized to the maximum value (Val[101] $C^{\gamma 2}$) and to the volumes of the corresponding ^{1}H–^{13}C cross-peaks in the 2D HSQC spectrum to compensate for relaxation and differential enrichment effects. The centroids of the methyl hydrogen positions were used to derive NOE volume predictions from the crystal structures of the wild-type protein and G89P variants and then weighted at 79 % and 21 % respectively.

Table S1 Methyl carbon-centred NOE analysis of Trp[59] indole reorientation in FKBP12

Leu[74] was not included due to ambiguity in the stereochemical assignment of the methyl resonances. Interactions for methyl–$H^{N\epsilon 1}$ distances >7 Å were not included. The ratio of volumes between the normalized NOEs for PDB code 2PPN wild-type/G89P was 0.25. Distance, distance between methyl carbon to the indole $H^{N\epsilon 1}$ in the PDB code 2PPN wild-type and the G89P variant crystal structures. NOE(exp), normalized experimental volumes for the methyl–indole $H^{N\epsilon 1}$ NOESY cross-peaks.

Residue	Atom	NOE(exp)	$Distance_{2PPN}$ (Å)	NOE (S^2)	$Distance_{G89P}$ (Å)	NOE (S^2)
Ile[56]	$C^{\delta 1}$	0.795	4.38	0.713		
Ile[76]	$C^{\delta 1}$	0.828	4.14	1.000		
Val[55]	$C^{\gamma 2}$	0.048			6.59	0.015
Val[24]	$C^{\gamma 2}$	0.460			3.89	0.369
Val[101]	$C^{\gamma 1}$	0.650	6.82	0.050	3.37	0.860
Val[24]	$C^{\gamma 1}$	0.308			4.54	0.145
Val[101]*	$C^{\gamma 2}$	1.000	4.98	0.330	3.32	1.000
Val[63]	$C^{\gamma 2}$	0.651	6.88	0.047	3.60	0.579
Leu[97]	$C^{\delta 1}$	0.050	6.27	0.082		

*This cross-peak also includes the degenerate Val[63] $C^{\gamma 1}$ which contributes only 4 % of the predicted volume.

© 2014 The Author(s)
The author(s) has paid for this article to be freely available under the terms of the Creative Commons Attribution Licence (CC-BY) (http://creativecommons.org/licenses/by/3.0/) which permits unrestricted use, distribution and reproduction in any medium, provided the original work is properly cited.

Table S2 Methyl proton centroid NOE analysis of Trp[59] indole reorientation in FKBP12

Interactions for methyl–$H^{N\varepsilon 1}$ distances >7 Å were not included. The ratio of volumes between the normalized NOEs for PDB code 2PPN wild-type/G89P was 0.37. Distance, distance between methyl carbon to the indole $H^{N\varepsilon 1}$ in the PDB code 2PPN wild-type and the G89P variant crystal structures. NOE(exp), normalized experimental volumes for the methyl–indole $H^{N\varepsilon 1}$ NOESY cross-peaks.

Residue	Atom	NOE(exp)	$Distance_{2PPN}$ (Å)	NOE (S^2)	$Distance_{G89P}$ (Å)	NOE (S^2)
Ile[56]	$C^{\delta 1}$	0.795	4.37	0.442		
Ile[76]	$C^{\delta 1}$	0.828	3.82	1.000		
Val[55]	$C^{\gamma 2}$	0.048			6.64	0.013
Val[24]	$C^{\gamma 2}$	0.460			3.80	0.384
Val[101]	$C^{\gamma 1}$	0.650	6.96	0.027	3.24	1.000
Val[24]	$C^{\gamma 1}$	0.308			4.61	0.121
Val[101]*	$C^{\gamma 2}$	1.000	4.63	0.315	3.32	0.910
Val[63]	$C^{\gamma 2}$	0.651	6.56	0.039	3.28	0.931
Leu[97]	$C^{\delta 1}$	0.050	6.12	0.059		

*This cross-peak also includes the degenerate Val[63] $C^{\gamma 1}$ which contributes only 4 % of the predicted volume.

REFERENCE

1 Hernández, G. and LeMaster, D. M. (2001) Reduced temperature dependence of collective conformational opening in a hyperthermophile rubredoxin. Biochemistry **40**, 14384–14391

Received 29 October 2013/17 December 2013; accepted 10 January 2014
Published as BJ Immediate Publication 10 January 2014, doi:10.1042/BJ20131429

© 2014 The Author(s)
The author(s) has paid for this article to be freely available under the terms of the Creative Commons Attribution Licence (CC-BY) (http://creativecommons.org/licenses/by/3.0/) which permits unrestricted use, distribution and reproduction in any medium, provided the original work is properly cited.
